# Efficacy and safety of Dengyinnaotong Capsule in patients with Cognitive impairment caused by cerebral Small Vessel Disease: study protocol of a multicenter, randomized, open-label, controlled trial (De-CSVD trial)

**DOI:** 10.1186/s13063-022-06646-6

**Published:** 2022-08-17

**Authors:** Yan-Xia Li, Jin-Cun Li, Min Tian, Mao-Yong Zheng, Li-Ping Zhang, Jin-Lu Zhang, Feng Yu, Yi-Zhao Li, Qing-Hua Zhang

**Affiliations:** 1 Department of Neurology, Shandong Second Provincial General Hospital, Jinan, Shandong China; 2 Department of Administration, Shandong Second Provincial General Hospital, Jinan, Shandong China; 3 Department of Neurology, Jinan Fanggan Rehabilitation Hospital, Jinan, Shandong China; 4 Department of Neurology, Shandong Second Provincial General Hospital, Shandong University, Jinan, Shandong China

**Keywords:** Cerebral small vessel disease, Cognitive function, Dengyinnaotong Capsule, Study protocol

## Abstract

**Background:**

Cerebral small vessel disease (CSVD) is a common syndrome in the older population, with a prevalence ranging from 5% in subjects aged 50 years to almost 100% in those aged 90 years and older. It is regarded to be a major cause of vascular cognitive impairment. Existing prevention and treatment approaches have not yet shown ideal clinical outcomes. Dengyinnaotong Capsule has shown great potential for improving cognitive function. This trial (De-CSVD trial) is designed to investigate the efficacy and safety of Dengyinnaotong Capsule on cognitive function in patients with CSVD .

**Methods:**

This multicenter, randomized, open-label, controlled trial is planned to recruit at least 270 patients with mild cognitive impairment related to CSVD in 25 centers in China. Recruitment started on 10 May 2021 and is foreseen to end on 31 December 2022. The final follow-up of participants will be completed by the end of March 2023. Participants will be randomized in a ratio of 1:1 to the experimental group (routine basic treatment plus Dengyinnaotong Capsule) or the control group (routine basic treatment). The primary outcome is the change in the Montreal Cognitive Assessment score from baseline to week 12. Secondary outcomes are changes in Shape Trail Test, Activities of Daily Living, Geriatric Depression Scale, and Dizziness Handicap Inventory score from baseline to week 12, new vascular events, and the changes in serum level of homocysteine, high-sensitivity C-reactive protein, and D-dimer from baseline to week 4 and 12, respectively. The exploratory outcome is the changes in the Tinetti performance-oriented mobility assessment score from baseline to week 12. Safety assessment is performed by monitoring vital signs, general biochemical examinations, 12-lead electrocardiogram examinations, and incidence of cardiovascular and cerebrovascular ischemia or bleeding events. Visits will be performed at week 0 (baseline, pre-randomization), week 4, and week 12 in the treatment period (post-randomization).

**Discussion:**

This trial is the first to investigate the efficacy and safety of Dengyinnaotong Capsule on cognitive impairment in patients with CSVD. The findings of this study might provide convincing evidence regarding the efficacy of Dengyinnaotong Capsule in patients with mild cognitive impairment related to CSVD.

**Trial registration:**

Chinese Clinical Trial Registry ChiCTR2100045831. Registered on 25 April 2021.

## Background

Cerebral small vessel disease (CSVD) is a quite common syndrome in the older population, with a prevalence ranging from 5% in subjects aged 50 years to almost 100% in those aged 90 years and older [1]. It affects cerebral small vessels composed of capillaries, small arteries, arteries, venules, and small veins [2]. In the field of neuroimaging, the CSVD is marked by enlarged perivascular spaces, small subcortical infarcts, lacunes, white matter hyperintensities, cerebral microbleeds, and brain atrophy [3]. The prevalence of lacunes, white matter hyperintensities, and microbleeds in the elderly population is around 8–28%, 50–98%, and 5%, respectively [4]. In recent years, CSVD has attracted more and more attention and is regarded to be one of the main causes of vascular cognitive impairment [5, 6]. Vascular cognitive impairment is considered a gradual process from normal cognitive status to mild cognitive impairment and then dementia [7]. With the steady growth of life expectancy worldwide, the incidence rate of age-related CSVD is also increasing, leading to a high global burden of stroke and vascular dementia [8, 9]. Up to now, a healthy lifestyle, modification of traditional risk factors (such as hypertension, hypercholesterolemia, diabetes mellitus, and smoking), cholinesterase inhibitors, memantine, and selective calcium channel blockers with mainly vascular effects are still the most important preventive and therapeutic approaches for CSVD [10, 11]. However, most of these conventional treatments were limited by the frequent contraindications and adverse effects (AEs), high drug burden, and dissatisfied clinical outcomes. Therefore, developing novel treatment strategies is quite important for patients with CSVD.

Dengyinnaotong Capsule is one of the traditional Chinese medicine recipes for promoting blood flow and dispelling wind-evil. It is modified from traditional Yi medicine and consists of four herbs (*Erigeron breviscapus*, *Leaves Ginkgo biloba*, *Gaultheria cumingiana*, and *Radix Panax notoginseng*). Several studies have proved the role of the Dengyinnaotong Capsule in improving cognitive function. A preclinical study has shown that Dengyinnaotong Capsule significantly improves the learning and memory function of an aging model mouse [12]. Chen et al. have found that Dengyinnaotong Capsule combined with Naoxintong Capsule can significantly improve the nerve function and life ability of patients with cerebral infarction in the convalescent phase when compared to Naoxintong Capsule [13]. Wu et al. compared the efficacy of Dengyinnaotong Capsule and Yinxingye Capsule on the treatment of patients with ischemic stroke in the convalescent and sequelae phase [14]. The study showed that Dengyinnaotong Capsule is better than Yinxingye Capsule in terms of effective rate and improvement symptoms in the treatment of ischemic stroke. All the above evidence suggests that Dengyinnaotong Capsule might be a promising approach for treating patients with mild cognitive impairment related to CSVD.

Therefore, a multicenter, randomized, open-label, controlled trial (De-CSVD trial) is designed to investigate the efficacy and safety of Dengyinnaotong Capsule on cognitive function in patients with CSVD . We hypothesize that Dengyinnaotong Capsule plus routine basic treatment is more effective in terms of improving cognitive impairment when compared with routine basic treatment. Herein, the methods used in the ongoing De-CSVD trial are described.

## Methods/design

### Study design

**Fig. 1:**
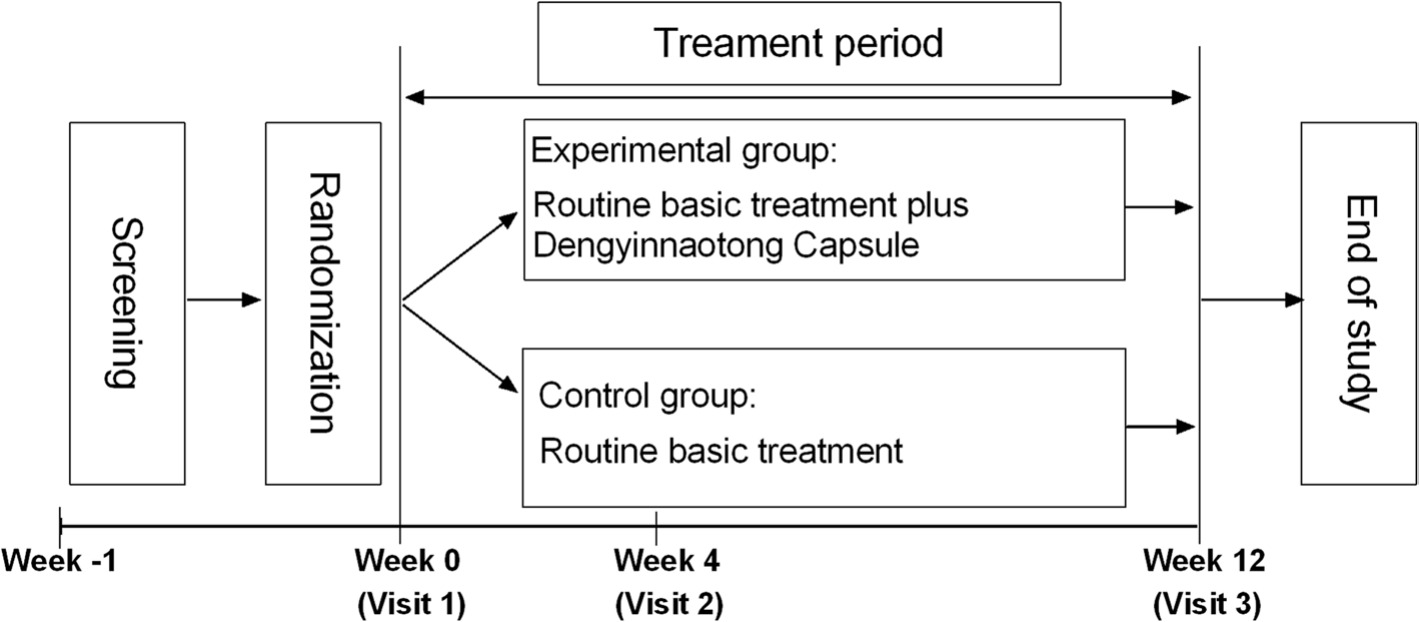
Study design

De-CSVD is designed as a multicenter, randomized, open-label, parallel-group, exploratory trial to determine the efficacy and safety of Dengyinnaotong Capsule on cognitive function in patients with CSVD . Recruitment started on 10 May 2021 and is foreseen to end on 31 December 2022. After a screening period within 7 days, eligible participants will be randomized to the experimental group (routine basic treatment plus Dengyinnaotong Capsule) or the control group (routine basic treatment) in a 1:1 ratio. Visits will be performed at weeks 0, 4, and 12 in the treatment period. This trial was designed according to the SPRIT 2013 Statement [15]. An overview of the study design is shown in Fig. 1.

### Trial setting

Participants will be recruited from 25 centers of Shandong province in China, including Shandong Second Provincial General Hospital, Heze Municipal Hospital, Affiliated Hospital of Heze Medical College, Affiliated Hospital of Jining Medical University, Dezhou People’s Hospital, Dezhou Seventh People’s Hospital, Dezhou Hospital of Traditional Chinese Medicine, Heze Mudan People’s Hospital, Heze Second People’s Hospital, Caoxian People’s Hospital, The Second Affiliated Hospital of Shandong First Medical University, Laizhou Hospital of Traditional Chinese Medicine, Binzhou Second People’s Hospital, Yucheng People’s Hospital, Wenshang People’s Hospital, Linyi Luozhuang District People’s Hospital, Zhanhua Traditional Chinese Medicine Hospital of Binzhou City, Gaomi Municipal Hospital, Dezhou Zhaoxing Hospital, Chengwu Social Welfare Center, Shandong Yinfeng Institute of Life Sciences, Jinan Fanggan Rehabilitation Hospital, Jinan Fuyuan Rehabilitation Hospital, Dongming Sunshine Rehabilitation Hospital, Jinjiang Qingniao Shijia Rehabilitation Hospital, and Zhongshan Rehabilitation Branch of Jiangsu Provincial People’s Hospital.

### Participant recruitment

Physicians in the 25 centers are approached and informed about the trial. Physicians will identify potentially eligible patients at their practice using an automated search. Potentially eligible patients will receive a preliminary participant information sheet about the trial. Consent to enter the trial will be sought only after a full explanation has been given and time allowed for consideration. Patients who are willing to participate in the trial will be asked to sign a consent form. If the patient lacks the capacity to provide informed consent, a legal representative will be asked to provide consent. Consent will be taken by a research nurse or a registered medical professional with suitable study training, as delegated by the principal investigator at each center.

### Participants eligibility

The inclusion criteria of participants are as follows: (1) participants aged 50–70 years; (2) participants with cognitive decline in memory or other cognitive domains at least for 3 months, and was diagnosed with mild cognitive impairment (cognitive decline in one or more cognitive domains and is insufficient to affect the independence of life) according to the “Chinese Diagnosis and Treatment Guidelines for Cognitive Dysfunction Related to Cerebral Small Vascular Disease (2019)” [16]; (3) participants with the Montreal Cognitive Assessment (MOCA) score<26 (does not meet the diagnostic criteria of dementia according to the Diagnostic and Statistical Manual of Mental Disorders, fourth edition [17]) and a clinical dementia rating scale (CDR) score of 0.5 points [18]; (4) head computerized tomography or magnetic resonance imaging showed recent small subcortical infarcts, lacunes, white matter hyperintensities (WMHs), enlarged perivascular spaces, and microbleeds, and according to the “Chinese Diagnosis and Treatment Guidelines for Cognitive Dysfunction Related to Cerebral Small Vascular Disease (2019)” and “Consensus on Diagnosis and Treatment of Cerebral Small Vascular Disease in China (2015)” [19] (more than 2 lacunar infarcts outside the brain stem, Fazekas score ≥ 2 and number of cerebral micro hemorrhage ≥ 3); and (5) participants with signed informed consent for trial participation.

Exclusion criteria are as follows: (1) participants with intracranial hemorrhage or massive hemorrhage of other organs; (2) participants with a history of severe mental illness or epilepsy; (3) participants with a history of new stroke within 3 months before admission; (4) participants with a history of alcohol or drug abusers; (5) participants with Alzheimer’s disease, Lewy body dementia, frontotemporal lobe dementia, brain tumors, hydrocephalus or other central nervous diseases; (6) participants who are unable to complete neuropsychological test and magnetic resonance imaging examination; (7) participants who had participated in other drug clinical trials as subjects in the past 30 days; and (8) researchers consider it impossible for the subject to complete the study.

Withdrawal criteria from the treatment are as follows: (1) participants or their guardian withdraw informed consent; (2) participants lost to follow-up; (3) sensitivity to Dengyinnaotong Capsule or their components; (4) participants experience severe complications or deterioration during the trial period in which urgent measures were required; (5) participants become pregnant during the trial; and (6) other reasons. The reason for the participant being withdrawn from the trial will be recorded. If the participant is still willing to complete the follow-up, then the follow-up process will continue.

### Sample size calculation

The sample size calculation is based on previous studies [14, 20, 21]. The routine basic treatment has been proved to improve the MOCA score of patients with CSVD by 2 points [20]. Besides, the routine basic treatment combined with Yinxingye is effective in the treatment of cognitive impairment in non-demented patients, with a MOCA score improved by 3 points [21]. Moreover, Wu et al. have found that Dengyinnaotong Capsule is better than Yinxingye Capsule in terms of effective rate and improvement symptoms in the treatment of ischemic stroke [14]. Therefore, based on the above results, we assume that the routine basic treatment combined with Dengyinnaotong Capsule in this trial will improve the MOCA score of patients with CSVD by 2 points compared with the routine basic treatment. Accordingly, 108 per group will be sufficient with a level of significance of 95% (*α*=0.05), a power of 90% (*β*=0.1), and an assumed standard deviation (SD) of 4.5. Considering the drop-out rate of 20%, a total of 270 samples (135 per group) are needed at least.

### Randomization, allocation concealment, and blinding

All eligible participants are randomly assigned to an experimental group (routine basic treatment plus Dengyinnaotong Capsule) or control group (routine basic treatment) in a ratio of 1:1. The simple randomization is generated centrally by the trial biostatistician using a computer-generated random number table. A central, web-based system will provide a unique trial identifier for each participant via email to a delegated member of site staff. Due to the open-label design, the participant, investigator, and project management teams are aware of the treatment allocation.

### Intervention

Participants in the control group are treated with routine basic treatment. Participants in the experimental group are treated with routine basic treatment plus Dengyinnaotong Capsule (Kunming Pharmaceutical Group Co., Ltd., Yunnan, China; 0.52 g/time, 3 times/day). The routine basic treatment is performed according to the “Chinese Diagnosis and Treatment Guidelines for Cognitive Dysfunction Related to Cerebral Small Vascular Disease (2019)” and “Consensus on Diagnosis and Treatment of Cerebral Small Vascular Disease in China (2015)” [19]. The total treatment period is 12 weeks. If there are AEs during treatment, the investigators can decide whether to terminate or postpone the medication according to the severity. In addition, for other concomitant diseases, combined drugs can be used on the premise of not affecting the judgment of the efficacy of the test drugs. During the trial period, it is forbidden to use other Chinese medicines, including Chinese patent medicine, Chinese medicine injections, and Chinese medicine decoctions, decoction pieces, and formula granules.

During the trial, to ensure the compliance of participants, it is important for them to fully understand the significance of the trial, the importance of taking the medicine on time, and the necessity for follow-up. The subjects will be required to take the medicine according to the regulations, fill in the patient diary card on time, and attend the follow-up as required. In order to assess drug compliance, participants will be checked for the remaining number of drugs at each follow-up visit.

### Data collection

In this trial, data collection will be performed at weeks 0, 4, and 12 in the treatment period. The schedule of data collection is shown in Table 1. General biochemical examinations include blood routine, urine routine, stool routine, coagulation function, liver function, renal function, blood glucose, and blood lipid examination.

**Table 1:** Schedule of data collection

Visits	Screening and baseline	Treatment period	
	0	1	2
Time points (weeks)	− 1 to 0	4	12
Time windows (days)	-	±7	±7
Informed consent	×		
Inclusion and exclusion criteria	×		
History of diagnosis and treatment	×		
Vital signs and physical examinations	×	×	×
General biochemical examinations	×	×	×
12-lead electrocardiogram examination	×	×^a^	×
CT or MRI examination	×	×^a^	×^a^
MoCA score	×		×
CDR score	×		×
Shape Trail Test	×	×^a^	×
ADL score	×		×
DHI score	×^a^	×^a^	×^a^
Tinetti POMA score	×^a^	×^a^	×^a^
GDS score	×^a^	×^a^	×^a^
Serum levels of Hcy, HS-CRP, and D-dimer	×	×	×
Audiological examination	×^a^	×^a^	×^a^
Drug combination	×	×	×

*MoCA* Montreal Cognitive Assessment, *CDR* Clinical Dementia Rating, *CT* Computed tomography, *MRI* Magnetic resonance imaging, *ADL* Activities Of Daily Living, *DH* Dizziness Handicap Inventory, *Tinetti POMA* Tinetti performance-oriented mobility assessment, *GDS* Geriatric Depression Scale, *Hcy* homocysteine, *HS-CRP* High-sensitivity C-reactive protein

^a^For patients with relevant clinical manifestations or willing to be examined

The 30-item clinician-administered MoCA is a brief cognitive screening tool with high sensitivity for screening patients with mild cognitive impairment [22]. It is a simple 10-min paper-and-pencil test that assesses multiple cognitive domains including memory, attention, concentration, language, visuospatial skills, abstraction, calculation, executive functions, and orientation.

The clinician-administered Shape Trail Test (STT) measures the ability to shift attention and visual attention [23]. STT part A requires test-takers to connect numbers (1–25) that are randomly distributed on a test paper (1-2-3 … ). In STT part B, a number (1–25) is surrounded by either a circle or a square, and the test-takers are asked to connect the numbers in a sequence. Once the test-taker places the pen or pencil on the first circle, the researcher will start recording the time and stop measuring the time when the pencil touches the last number. Taking the sum of completion time as the scoring standard (the shorter the time, the better the executive function) [24].

The Barthel index is a widely used tool for evaluating physical performance in Activities of Daily Living (ADL) [25]. The Barthel index includes ten items in two major areas of self-care activities (feeding, bathing, grooming, dressing, bowel and bladder control, and toileting) and mobility (including mobility, transfer, and using stairs). The score ranges from 0 (disabled) to 100 (very independent). The self-perceived level of handicap associated with the symptom of dizziness is measured by the 25-item Dizziness Handicap Inventory (DHI) [26]. Both ADL and GHI are self-report questionnaires.

The self-reported Tinetti performance-oriented mobility assessment (POMA) examines the level of mobility and balance to determine the degree of fall risk in the elderly [27]. This examination tool consists of the mobility subscale (8 items, 12 points) and balance subscale (9 items, 16 points), totaling 28 points. A higher score reflects a better functioning of the patient in mobility and balance. Depression in geriatric populations is assessed by the self-reported Geriatric Depression Scale (GDS) [28]. It is designed as a 30-item inventory with a yes/no format. A GDS score of 10 or fewer is considered normal, 11 to 20 is considered mildly depressed, and 21 or more means moderately to severely depressed.

### Outcomes

The primary outcome is the change in MoCA score from baseline to week 12. The secondary outcomes include the following: (1) the changes in STT, ADL, GDS, and DHI score from baseline to week 12; (2) new vascular events; and (3) the changes in serum level of homocysteine (Hcy), high-sensitivity C-reactive protein (HS-CRP), and D-dimer from baseline to week 4 and 12, respectively. The exploratory outcome is the changes in the Tinetti POMA score from baseline to week 12.

### Safety assessment

Safety assessment is performed at weeks 0, 4, and 12 by monitoring vital signs, general biochemical examinations, 12-lead electrocardiogram examinations, and the incidence of cardiovascular and cerebrovascular ischemia or bleeding events. All AEs observed by investigators or reported spontaneously by participants throughout the trial will be registered. For related to the trial, the investigators will give symptomatic treatment. Investigators will also collect the detailed characteristics of AEs, including reasons, grades, actions taken regarding AEs, dates of treatment initiation and end, and treatment outcomes. AEs are graded as mild, moderate, serious, life-threatening, and death. Serious AEs (SAEs) include death, a life-threatening AE, hospitalization or prolongation of hospitalization, a persistent or significant disability, a congenital anomaly, or a significant medical event that requires intervention. Any SAEs should be reported to the local Food and Drug Administration, National Medical Products Administration, and National Health and Family Planning Commission within 24 hours.

### Plans to promote participant retention and complete follow-up

Participants will receive extensive information about the study setup and requirements during the recruitment process. The importance of completing the follow-up will be stressed. The trial will maximize both enrollment and participant retention via multiple pathways, including collecting support system contact information during informed consent, appointment reminders, and support for transportation. Participants will be allowed to withdraw from treatment at any time. Participants who prematurely exit the study will still be encouraged to participate in regular follow-up visits, ensuring data will continue to be collected.

### Statistical analysis

The efficacy analysis is based on the intention-to-treat (ITT) population, which is defined as all randomized participants. All efficacy analysis results are analyzed in the full analysis set (FAS) and per-protocol set (PPS). The FAS is defined as all randomized patients who received at least one dose of the study drug and had both baseline and at least one post-baseline measurement of primary efficacy. The PPS is defined as all patients who completed the treatment without serious violation of the protocol. The safety analysis is performed in a safety set (SS), which is defined as participants who receive at least one dose of the study drug and at least one assessment of safety data. Categorical data will be expressed as numbers and percentages, and quantitative data will be expressed as means±SD, median, interquartile range (IQR) values, or minimum and maximum. For the primary outcome, the analysis of covariance (ANCOVA) will be performed to compare the 12-month MoCA outcomes (the change in MoCA score from baseline to week 12) between the groups at follow-up adjusting for baseline scores. For secondary and exploratory outcomes, the ANCOVA will also be performed to test for statistical differences between the groups. A repeated-measures analysis of variance (ANOVA) was performed to investigate the time-course of the changes in serum level of Hcy, HS-CRP, and D-dimer from baseline to weeks 4 and 12 within each group and to compare those between two groups. Pearson *χ*^2^ or Fisher’s exact test will be performed to compare differences in AEs of two groups. Any potential effects related to site differences will be assessed using mixed models. The baseline will be taken as the covariate in the model, and the effect of the center will be considered. In the case of missing data, we will capitalize on multiple imputation techniques to handle the issue of missing data in this study. All statistical analyses will be performed using Statistical Analysis System (SAS Institute, Inc, Gary, NC), and statistical significance was set at *p*<0.05.

### Register, ethics, and data management

This study has been prospectively registered on 25 April 2021 at the Chinese Clinical Trial Registry (ChiCTR2100045831). This trial is conducted by the Declaration of Helsinki and Good Clinical Practice Guidelines and approved by the ethics committee institutional review board of each participating center. Any change to the protocol will be approved by the relevant regulatory authorities and require participants to re-consent before continuing in the study. Data were collected on paper first, and then is being entered and managed by a designated Data Manager using EpiData software (EpiData 3.01 for Windows; The EpiData Association, Odense, Denmark). To ensure the accuracy of the data, two data managers will independently carry out data entry and proofreading. The regular oversight will be conducted by an experienced Clinical Research Associate by monitoring the quality, safety, and progress of the study. The database will be locked post reconciliation of all data. The locked database will be provided to the statisticians to conduct the independent statistical analysis. There are no interim analyses planned. Study findings will be disseminated through peer-reviewed publications and presentations at scientific meetings.

## Discussion

CSVD refers to a syndrome that is characterized by the damage to white and deep grey matter structures of the brain [7]. It is estimated that more than 73% of middle-aged subjects suffer from CSVD, of which 9% are severe CSVD (defined as high white matter hyperintensity burden, lacunar infarction, or microhemorrhage) [29]. Higher white matter hyperintensity burden is related to cognitive dysfunction, and severe CSVD is one of the main risk factors for mild cognitive impairment [29]. Without timely intervention, mild cognitive impairment will eventually lead to moderate to severe dementia [30]. Given that existing prevention and treatment approaches have not yet shown ideal long-term outcomes, developing novel treatment strategies is quite important for patients with CSVD. As a traditional Chinese medicine recipe, Dengyinnaotong Capsule has shown great potential for promoting blood flow, dispelling wind-evil, and improving cognitive function [12–14]. However, the role of Dengyinnaotong Capsule on mild cognitive impairment related to CSVD is unclear. Therefore, a multicenter, randomized, open-label, controlled trial is designed to investigate the efficacy and safety of Dengyinnaotong Capsule on cognitive function in patients with CSVD for the first time.

This trial has several strengths. First of all, this study is designed as a multicenter, randomized, controlled trial. Participants will be recruited from 25 centers in China to ensure the representation of samples. Secondly, this trial focus on participants aged 50–75 years, among which the risks of stroke and dementia are higher [31]. Thirdly, the study outcomes are measured by using both clinician-administered and self-reported methods through a series of psychometric tools, which ensures the availability of good quality data. Fourthly, the data will be managed under the oversight of an independent Clinical Research Associate, which is critical to the credibility of future findings of this trial.

Despite the above strengths, there are still some limitations in the present study. Firstly, due to the different administration dosages of the two groups, the trial is openly labeled. The lack of blinding has the risk of both performance and detection bias. For example, patients or investigators might add concomitant treatments to address insufficient efficacy or manage symptoms or risk based on their beliefs and knowledge about treatment allocation. Secondly, although the vascular cognitive impairment is a gradual process from normal cognitive status to mild cognitive impairment and then dementia, this study only assesses the 12-week treatment outcome and does not seek to assess the long-term efficacy considering the poor compliance among elderly patients. Finally, we only evaluated the efficacy and safety of Dengyinnaotong Capsule on cognitive function related to CSVD at the specific dose. Dose-exploration study should be carried out in the further to determine the optimal dose of Dengyinnaotong Capsule for treating mild cognitive impairment related to CSVD.

In summary, the present trial is the first to investigate the efficacy and safety of Dengyinnaotong Capsule on cognitive function in patients with CSVD. The findings of this study might provide convincing evidence regarding the efficacy of Dengyinnaotong Capsule on mild cognitive impairment related to CSVD.

### Trial status

Recruitment started on 10 May 2021 and is foreseen to end on 31 December 2022.

## Data Availability

The datasets used and/or analyzed during the current study are available from the corresponding author on reasonable request.

## Abbreviations

AEs: Adverse effects
MOCA: Montreal Cognitive Assessment
CDR: Clinical dementia rating scale
WMHs: White matter hyperintensities
ADL: Activities of Daily Living
POMA: Performance-oriented mobility assessment
GDS: Geriatric Depression Scale
Hcy: Homocysteine
HS-CRP: High-sensitivity C-reactive protein
SAEs: Serious AEs
ITT: Intention-to-treat
FAS: Full analysis set
PPS: Per-protocol set
SS: Safety set
SD: Standard deviations
IQR: Interquartile range
SAS: Statistical Analysis System
